# Using FRAME to characterize provider‐identified adaptations to a stepped care intervention for adolescents and youth living with HIV in Kenya: a mixed methods approach

**DOI:** 10.1002/jia2.26261

**Published:** 2024-07-05

**Authors:** Nok Chhun, Dorothy Oketch, Kawango Agot, Dorothy I. Mangale, Jacinta Badia, James Kibugi, Wenwen Jiang, Mary Kirk, Barbra A. Richardson, Pamela K. Kohler, Grace John‐Stewart, Kristin Beima‐Sofie

**Affiliations:** ^1^ Department of Global Health University of Washington Seattle Washington USA; ^2^ Impact Research and Development Organization Kisumu Kenya; ^3^ Department of Oncology Washington University St. Louis Missouri USA; ^4^ Department of Epidemiology University of Washington Seattle Washington USA; ^5^ Department of Biostatistics University of Washington Seattle Washington USA; ^6^ Department of Child, Family, and Population Health Nursing University of Washington Seattle Washington USA; ^7^ Department of Pediatrics University of Washington Seattle Washington USA; ^8^ Department of Medicine University of Washington Seattle Washington USA

**Keywords:** adaptation, adolescent and youth, continuous quality improvement, FRAME, HIV, implementation science

## Abstract

**Introduction:**

The Data‐informed Stepped Care (DiSC) study is a cluster‐randomized trial implemented in 24 HIV care clinics in Kenya, aimed at improving retention in care for adolescents and youth living with HIV (AYLHIV). DiSC is a multi‐component intervention that assigns AYLHIV to different intensity (steps) of services according to risk. We used the Framework for Reporting Adaptations and Modifications‐Expanded (FRAME) to characterize provider‐identified adaptations to the implementation of DiSC to optimize uptake and delivery, and determine the influence on implementation outcomes.

**Methods:**

Between May and December 2022, we conducted continuous quality improvement (CQI) meetings with providers to optimize DiSC implementation at 12 intervention sites. The meetings were guided by plan‐do‐study‐act processes to identify challenges during early phase implementation and propose targeted adaptations. Meetings were audio‐recorded and analysed using FRAME to categorize the level, context and content of planned adaptations and determine if adaptations were fidelity consistent. Providers completed surveys to quantify perceptions of DiSC acceptability, appropriateness and feasibility. Mixed effects linear regression models were used to evaluate these implementation outcomes over time.

**Results:**

Providers participated in eight CQI meetings per facility over a 6‐month period. A total of 65 adaptations were included in the analysis. The majority focused on optimizing the integration of DiSC within the clinic (83%, *n* = 54), and consisted of improving documentation, addressing scheduling challenges and improving clinic workflow. Primary reasons for adaptation were to align delivery with AYLHIV needs and preferences and to increase reach among AYLHIV: with reminder calls to AYLHIV, collaborating with schools to ensure AYLHIV attended clinic appointments and addressing transportation challenges. All adaptations to optimize DiSC implementation were fidelity‐consistent. Provider perceptions of implementation were consistently high throughout the process, and on average, slightly improved each month for intervention acceptability (β = 0.011, 95% CI: 0.002, 0.020, *p* = 0.016), appropriateness (β = 0.012, 95% CI: 0.007, 0.027, *p*<0.001) and feasibility (β = 0.013, 95% CI: 0.004, 0.022, *p* = 0.005).

**Conclusions:**

Provider‐identified adaptations targeted improved integration into routine clinic practices and aimed to reduce barriers to service access unique to AYLHIV. Characterizing types of adaptations and adaptation rationale may enrich our understanding of the implementation context and improve abilities to tailor implementation strategies when scaling to new settings.

## 1 INTRODUCTION

Ending the HIV epidemic requires achieving UNAIDS 95‐95‐95 HIV care cascade targets [1, 2]. The greatest gap for adolescents and youth living with HIV (AYLHIV) along the care and treatment cascade is achieving the third 95 target, with greater than 30% of AYLHIV not achieving viral suppression after 12 months on treatment [3, 4]. Improving HIV care for AYLHIV requires tailoring interventions [5, 6, 7, 8, 9] to their unique social, interpersonal and developmental needs. Stepped Care is an evidence‐based service delivery model, used in mental health to align individual needs with provided services [10], simplifying care for clients who are effectively managing their health and providing more intensive care for clients who need additional support. Stepped care approaches have been utilized in high‐income and resource‐constrained settings, including Kenya [11, 12], to improve health outcomes for adolescent, youth and adult populations [13, 14].

A stepped care approach was used to deliver services for AYLHIV enrolled in the Data‐informed Stepped Care (DiSC) to Improve Adolescent HIV Outcomes study [15]. The DiSC intervention is multi‐component and includes a clinical assessment tool that health providers use to assign AYLHIV to “steps” according to their level of need, with each step corresponding to evidence‐based interventions aimed at supporting AYLHIV with retention in care. DiSC components include differentiated care [16, 17, 18, 19] and psychosocial interventions (enhanced adherence and mental health counselling [20]), which are important patient‐level strategies recommended by the World Health Organization for addressing the clinical care needs of adolescents and young adults living with HIV.

To ensure evidence‐based approaches for AYLHIV can effectively support retention in care, interventions need to be acceptable, appropriate and feasible for local healthcare settings and practice. Health providers play a critical role in providing HIV care for AYLHIV. Provider‐identified adaptations of interventions may ensure HIV care services align with the needs of AYLHIV while simultaneously optimizing available resources [21, 22]. Adaptations, defined as thoughtful and deliberate changes made to programmes or interventions [23, 24], are often made during the dynamic process of implementation. However, the who, when, what, how and why adaptations are identified and prioritized during implementation are often not well documented, limiting knowledge dissemination from implementation experiences. Sharing of lessons learned is critical to inform integration and future scalability of evidence‐based interventions that support AYLHIV with retention in care and to maintain life‐long adherence to antiretroviral therapy.

In this paper, we aim to characterize intervention adaptations made by providers during the early phase implementation of an ongoing cluster‐randomized clinical trial (cRCT) in western Kenya [15] and examine how adaptations influenced intervention acceptability, appropriateness and feasibility.

## 2 METHODS

### 2.1 Study setting, population and design

This analysis was implemented during the DiSC study, conducted from April 2022 to August 2023. DiSC was an effectiveness‐implementation hybrid type I cRCT aimed to improve retention in care for AYLHIV in 24 HIV care clinics across Kisumu, Homabay and Migori counties in western Kenya [15]. Participating HIV care clinics were selected based on client volume (≥100 AYLHIV in care), use of electronic medical records (EMRs) and facility leadership interest. During early phase implementation, providers participated in continuous quality improvement (CQI) meetings to identify and prioritize adaptations that optimized DiSC implementation. We examined how adaptations influenced acceptability, appropriateness and feasibility, measures which serve as indicators of the implementation process and are critical preconditions for achieving the desired impact on clinical outcomes [25, 26].

### 2.2 The DiSC intervention

DiSC employs a Stepped Care delivery model [14], an evidence‐based service delivery strategy, to align the needs of AYLHIV, ages 10–24, with provided services. The overall primary trial outcome measure is retention in care, defined as a missed visit (within a 30‐day time period for any visit) and 12‐month loss to follow‐up [15]. Using a Stepped Care approach, AYLHIV with the highest need receive the highest intensity and frequency of care while allowing those effectively managing their HIV care to benefit from less intensive services. As part of DiSC, a clinical assessment tool is used by providers to identify AYLHIV at risk for loss to follow‐up and assign them to four care “steps” (differentiated care, standard of care, individual counselling or intensive support) [15]. The lowest care step (Step 1) aligns with differentiated service delivery strategies where HIV services include fast‐track pharmacy refills and longer visit intervals [16, 27]. AYLHIV in Step 2 receive standard of care with standard visit intervals. As AYLHIV step up, visit frequency and service intensity increases, including the provision of cognitive behavioural therapy (CBT) counselling sessions for AYLHIV who screened positive for depression, as determined by the Patient Health Questionnaire 2‐item (PHQ‐2) depression screening tool (Step 3). In the highest step (Step 4), clients with unsuppressed viral loads receive enhanced adherence counselling sessions and case management services including home visits. Adolescents and youth enrolled from control facilities received standard of care per Government of Kenya guidelines, which did not include the clinical assessment tool and referral approach, though some elements such as enhanced adherence counselling would be implemented for AYLHIV with viral non‐suppression [27, 28].

### 2.3 Data collection

Between May and December 2022, health providers participated in twice‐monthly CQI meetings using plan‐do‐study‐act (PDSA) processes to make planned adaptations to optimize intervention implementation at their specific HIV care clinic. PDSA cycles consisted of four interrelated processes: (1) Plan—providers identify a challenge and specific adaptation (change concept) to address the identified challenge; (2) Do—providers implement the proposed adaptation; (3) Study—providers evaluate the effect of the adaptation by reviewing improvement indicators and implementation experiences; and (4) Act—providers make a decision to either accept the adaptation as implemented, continue adaptation to improve implementation or abandon the adaptation and begin a new cycle [29]; learnings from one cycle guide subsequent cycles. After the initial CQI meeting, the short‐term impact of adaptations [30] on implementation outcomes of reach, adoption and fidelity (correct step assignment and step reassessment) were shared at the subsequent meeting [31], allowing providers to determine if the adaptation resulted in short‐term improvement [30], thereby informing their decision to adopt, adapt or abandon each tested adaptation.

Although 144 CQI meetings were planned (12 per facility), 96 were implemented because providers experienced challenges identifying adaptations to test during subsequent cycles. Providers found the original 2‐week interval insufficient to test adaptations and assess the effect given AYLHIV return‐to‐clinic appointment windows. As a result, after the fourth CQI meeting, every other meeting shifted to informal check‐ins with DiSC study research assistants (RAs) without identification of new adaptations. At the final CQI, all sites except one opted to use their meeting to discuss overall experiences with CQI meetings, summarize adaptations they tested and which were still being implemented, rather than identifying a final challenge to address.

CQI meetings were audio‐recorded and facilitated by RAs. RAs were trained nurses or clinical officers. RAs used standardized PDSA worksheets to record highlights from each CQI discussion. During CQI meetings, providers identified challenges experienced during implementation and proposed targeted adaptations to intervention materials, procedures or delivery to specifically address each identified challenge. Data were collected and managed using REDCap (Research Electronic Data Capture), a web‐based software platform hosted at the University of Washington [32, 33].

Prior to each CQI meeting, providers completed individual surveys that quantified their perceptions of intervention acceptability, appropriateness and feasibility measures over time using a Likert scale from 1 to 5 (1 = strong disagreement; 5 = strong agreement) [25]. The survey was first administered April 2022 post‐training but prior to starting the cRCT, and subsequently repeated every 2 weeks for 6 months for a total of 13 survey timepoints.

### 2.4 Data processing

CQI adaptations were analysed by two primary analysts (NC and DO) using the Framework for Reporting Adaptations and Modifications to Evidence‐based interventions (FRAME). FRAME allowed systematic evaluation of provider‐identified planned adaptations over time to understand how and why health providers adapted delivery of DiSC [24]. Analysts reviewed PDSA worksheets and listened to CQI meeting audio recordings to identify key challenges experienced, adaptations planned and rationale for selecting specific adaptations. Data informed development of CQI meeting summaries. At the conclusion of the adaptation period, one primary analyst (NC) extracted adaptation data from CQI meeting summaries and PDSA worksheets into an excel matrix containing FRAME constructs to categorize the level, context and content of planned adaptations and determine if adaptations were fidelity consistent. The matrix also tracked whether adaptations were carried out as planned, and the final decision providers made on the proposed adaptation (i.e. adopt, adapt or abandon). Adaptations were also categorized based on whether they represented one change idea or comprised multiple changes proposed as a single adaptation.

Analysts reviewed the adaptation matrix [34] independently and resolved differences in FRAME construct classification through discussion, referring to the audio files as needed, until reaching an agreement.

### 2.5 Statistical analysis

SankeyMATIC was used to develop a Sankey Diagram that illustrated the application of FRAME constructs [35]. Facility‐specific trends in provider perceptions of intervention acceptability, appropriateness and feasibility over time were visually displayed using scatterplots. Mixed effects linear regression models were used to evaluate provider perceptions of acceptability, appropriateness and feasibility of the intervention over time (measured at monthly intervals) with random effects for provider and facility (*p*‐value <0.05 was considered significant). Analysis was conducted using a complete case analysis approach and it was determined a priori that missing provider data was missing at random. Analyses were performed using the R statistical computing environment [36].

### 2.6 Ethical considerations

Research ethics approval was received from the Maseno University Ethical Review Committee (MUERC/00917/20), the Kenya National Commission for Science, Technology and Innovation (444824) and the University of Washington Institutional Review Board (STUDY00011096). All health providers gave written informed consent.

## 3 RESULTS

Health providers included nurses (*n* = 16) and clinical officers (*n* = 38; Table 1). The majority of providers were female (52%) and were a median age of 34 years (interquartile range [IQR]: 31–38). Providers reported a median of 5 years (IQR: 2–8) at their clinic, a median of 7 years (IQR: 5–9) providing care to people living with HIV of all ages and a median of 6 years (IQR: 5–9) providing care and treatment specifically to AYLHIV.

**Table 1:** Demographic characteristics of health providers across intervention facilities

Characteristic^a^	*N* (%) or Median [IQR]
Primary work location	
Comprehensive care clinic	48 (89)
Other^b^	6 (11)
Sex	
Male	26 (48)
Female	28 (52)
Age (years)	34 [31, 38]
Highest level of education: university/college	54 (100)
Healthcare provider classification	
Clinical officer	38 (70)
Nurse/nurse counsellor	16 (30)
No. of years at current clinic	5 [2, 8]
No. of years providing HIV care to PLHIV (all ages)	7 [5, 9]
No. of years providing HIV care to AYLHIV (ages 10–24 years)	6 [5, 9]

*Note*: The majority of health providers filled out a demographic survey prior to the start of DiSC trial implementation, with five providers filling out demographic information when continuous quality improvement meetings concluded; four providers are missing demographic information and not included in this table.

Abbreviations: AYLHIV, adolescents and youth living with HIV; DiSC, Data‐informed Stepped Care; IQR, interquartile range; PLHIV, people living with HIV.

^a^ *N* = 54.

^b^ Other category includes: prevention of vertical transmission, maternal child health and outpatient departments.

Providers participated in a total of 96 CQI meetings, with each site completing at least eight PDSA cycles. Each CQI meeting included a median of three providers (IQR: 2–4) and lasted a median duration of 33 minutes (IQR: 20–44). CQI meetings where providers did not identify an adaptation (*n* = 13), or where adaptations were research‐related (*n* = 18), were excluded, leaving a total of 65 CQI meetings included in this analysis. Research‐related adaptations usually occurred during earlier CQI sessions (i.e. meetings 1–3) and involved initial study activities such as consenting and enrolment, whereas later adaptations were related to implementation.

### 3.1 Adaptation characteristics

Provider‐identified adaptations were characterized using FRAME (Table 2). Of the 65 adaptations analysed, 26 were unique. Adaptations based on one change concept (55%, *n* = 36) were as common as adaptations that represented multiple change concepts proposed as a single adaptation (45%, *n* = 29).

**Table 2:** Framework for Reporting Adaptations and Modifications‐Expanded (FRAME) classification system^a^ to characterize provider‐identified adaptations

Constructs	Coding categories
**WHEN** was the adaptation made?^b^	‐ During early implementation phase (initial 6 months)
Were adaptations PLANNED?^b^	‐ Planned/proactive with continuous quality improvement (CQI) using Plan‐Do‐Study‐Act (PDSA) cycles
**BY WHOM** are adaptations made?^b^	‐ Health providers (nurses and clinical officers) at intervention facilities
Consisted of ONE CHANGE IDEA or MULTIPLE CHANGE IDEAS proposed as one adaptation?^c^	‐ Single/Simple ‐ Bundled/Complex
**WHAT** was modified?	‐ Context (changes made to where, when, what or how) ‐ Content (changes made to content itself or impact how aspects of intervention are delivered) ‐ Training and evaluation (changes made to the way that staff are trained or how intervention is evaluated)
At what **LEVEL OF DELIVERY** (for whom/what are modifications made)	‐ Individual Recipient Level ‐ Target Intervention Group: all individuals with the problem that is being targeted ‐ Cohort/individuals that share a particular characteristic ‐ Individual Practitioner ‐ Specific Group of Practitioners ‐ Clinic/unit level (HIV clinic) ‐ Organization (entire Facility impacted) ‐ Network System/Community (outside Facility)
If CONTEXT changes, modifications are made to which of the following?	‐ Format ‐ Setting ‐ Personnel ‐ Population
If CONTENT changes, what was nature of modification?	‐ Tailoring/tweaking/refining ‐ Changes in packaging or materials ‐ Adding elements ‐ Removing/skipping elements ‐ Shortening/condensing (pacing/timing) ‐ Lengthening/extending (pacing/timing) ‐ Substituting ‐ Reordering of intervention modules or segments ‐ Spreading (breaking up session content over multiple sessions) ‐ Integrating the intervention into another framework ‐ Integrating another treatment into EBP ‐ Repeating elements or modules ‐ Loosening structure
	‐ Departing from the intervention (“drift”) followed by a return to protocol within the encounter ‐ Drift from protocol without returning
**WHY**? What was the goal of the adaptation?	‐ Increase reach (number of AYLHIV exposed or engaged with the intervention) ‐ Improve clinical effectiveness (improve clinical outcomes of AYLHIV) ‐ Increase adoption (number of providers using the DiSC tool/delivering intervention) ‐ Increase acceptability, appropriateness or feasibility (summed up as fit) ‐ Decrease costs of implementation ‐ Improve fidelity (correct step assignment) ‐ Improve fidelity (correct service exposure) ‐ Improve fidelity (re‐assessment of correct step assignment at subsequent visits) ‐ Improve fit with recipients ‐ Improve sustainability of EBP ‐ To address cultural factors
**LEVEL** of Rationale (Reasons for making the adaptation)	‐ Socio‐political ‐ Organization/Setting ‐ Provider ‐ Recipient/client
**FACTORS** that influenced level of rationale	‐ Socio‐political (existing laws, mandates, policies and regulations; political climate; societal/cultural norms) ‐ Organization/Setting (service structure; available resources; location/accessibility; time constraints) ‐ Provider (preferences; previous training and skills; perception of intervention) ‐ Recipient/client (motivation and readiness; access to resources)
Relationship to **FIDELITY**	‐ Fidelity Consistent/Core elements or functions preserved ‐ Fidelity Inconsistent/Core elements or functions changed ‐ Unknown
Adaptation **DECISION** ^c^	‐ Adopt ‐ Adapt ‐ Abandon

Abbreviations: AYLHIV, Adolescents and youth living with HIV; DiSC, Data‐informed Stepped Care; EBP, Evidence‐based practice.

^a^ Compiled from Stirman et al., 2019, FRAME coding manual, and Rabin et al., 2018, Table 2.

^b^ Not coded because no changes were made to these constructs during early phase implementation.

^c^ Additions to FRAME.

### 3.2 Contextual adaptations

**Figure 1:**
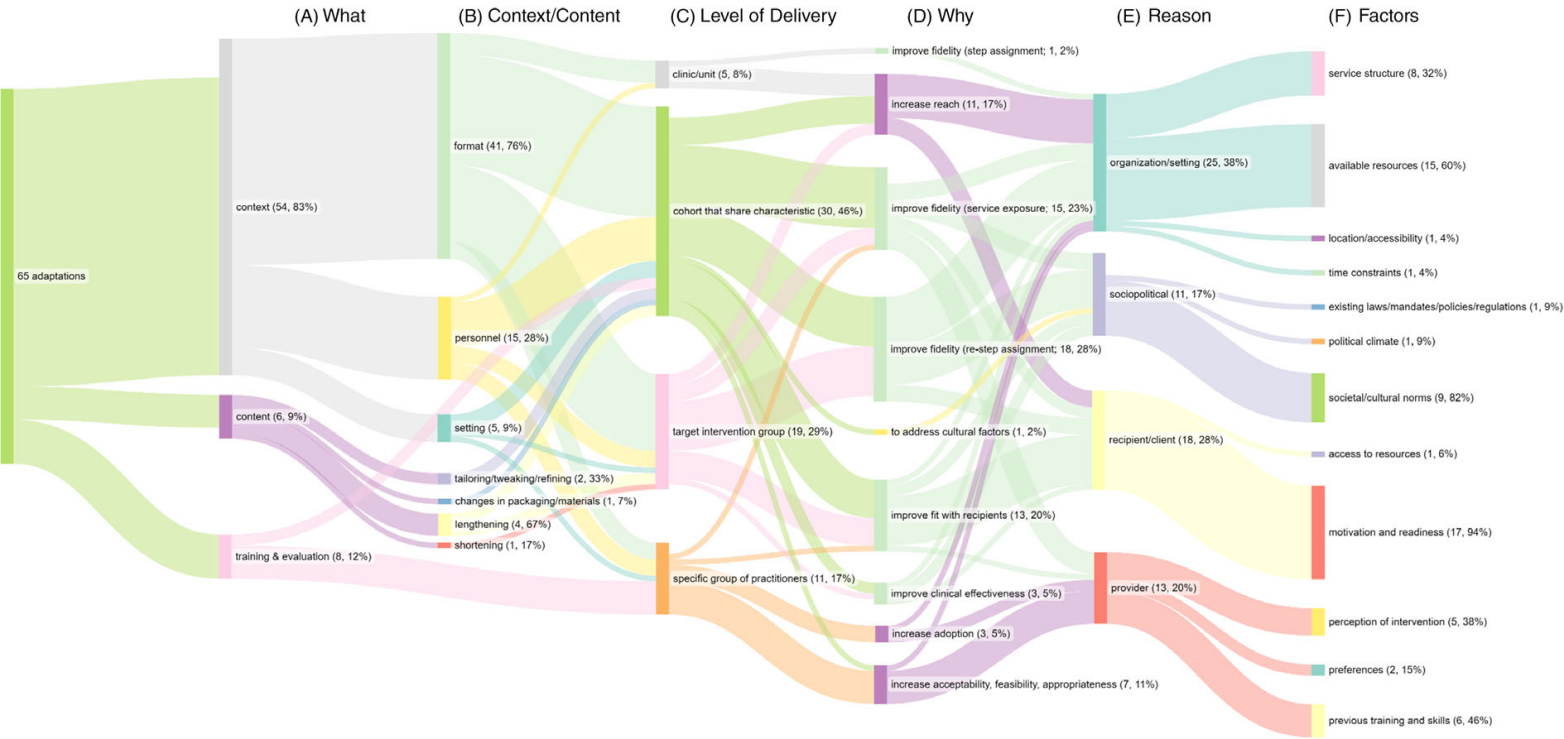
**Sankey Diagram for Framework for Reporting Adaptations and Modifications Expanded**. (A) WHAT was modified? (B) If context or content changes, modifications are made to which of the following? (C) At what LEVEL of delivery? (D) WHY? Goal of the adaptation. (E) LEVEL of rationale (reasons for making the adaptation). (F) FACTORS that influenced level of rationale. Adaptations may cover multiple categories, therefore, percentages may total more than 100%. Width of flow lines is related to percentage.

The majority of proposed adaptations were related to context (83%, *n* = 54), with 51 (78%) identified as exclusively context adaptations and three bundled with content‐related adaptations (Figure 1A). Context adaptations were made to format (76%, *n* = 41), personnel (28%, *n* = 15) and setting (9%, *n* = 5) to optimize how DiSC was implemented at each facility (Figure 1B). Format‐related context adaptations focused on the integration of DiSC within the clinic and consisted of: (1) improving clinic workflows to identify AYLHIV needing step reassessment at visit follow‐up; (2) decreasing AYLHIV waiting time at the clinic; (3) accommodating AYLHIV showing up for unscheduled visits; and (4) prioritizing specific clinic days for AYLHIV who need enhanced adherence counselling. Adaptations were also made to remind providers to use the paper‐based clinical assessment tool because the majority of facilities were using an EMR system. Adaptations also focused on improving documentation to ensure updated AYLHIV contact information for reachability for appointment reminders and mobile delivery of counselling sessions. Additionally, improving documentation facilitated AYLHIV attendance at clinic visits (rather than medication pick‐up by caregivers) by harmonizing visit schedules with school breaks.

Personnel‐related context adaptations included sensitizing other providers (ex: peer mentors, lab staff, adherence counsellors) to DiSC‐related service needs to streamline clinic flow and ensure access to data (e.g. uploading the latest viral load to the EMR to support step reassessment, updating AYLHIV contact information in the EMR). Setting‐related context adaptations were made to provide space, such as a tent or room, so that mental health screening and counselling sessions could be conducted in a confidential and private environment.

### 3.3 Content‐related adaptations

There were few targeted content‐specific adaptations (9%, *n* = 6; with three [5%] bundled with context‐related adaptations) given the focus on improving implementation rather than effectiveness. Content changes involved lengthening, shortening and tailoring/tweaking/refining AYLHIV return to clinic appointment schedules, even though this meant visit intervals were not aligned with the DiSC clinical assessment tool. These adaptations were made to harmonize clinic appointments with the school calendar, address safety concerns post‐presidential election results in 2022, address AYLHIV transportation challenges, and meet AYLHIV needs and preferences to have caregivers present during clinic appointments.

### 3.4 Training and evaluation adaptations

Adaptations related to training and evaluation (12%, *n* = 8) focused on improving documentation, and best practices regarding tracking and follow‐up of AYLHIV who were referred to mental health services to ensure receipt of motivational interviewing and CBT sessions, as recommended for AYLHIV assigned to the individual counselling “step.”

### 3.5 Adaptation rationale

Adaptations were primarily made to align intervention delivery with AYLHIV needs and preferences, prioritizing adaptations that improved the ability to meet the needs of specific groups of AYLHIV, such as those in boarding school or aged <15 years (46%, *n* = 30; Figure 1C). These findings align with the main goals of the adaptations which were to improve fidelity (step reassessment: 28%, *n* = 18; service exposure: 23%, *n* = 15; step assignment: 2%, *n* = 1), improve fit with AYLHIV (20%, *n* = 13), increase reach/engagement (17%, *n* = 11) and to improve clinical effectiveness (5%, *n* = 3; Figure 1D).

Adaptations most frequently targeted the organizational level (38%, *n* = 25; Figure 1E) and addressed resource availability (60%, *n* = 15) and service delivery structure (32%, *n* = 8; Figure 1F). For adaptations targeting the recipient level (28%, *n* = 18), the most frequent determinant was related to motivation and readiness (94%, *n* = 17). Adaptations targeted to the provider level (20%, *n* = 13) were mainly due to the provider's previous training and skills (46%, *n* = 6) and perception of the intervention (38%, *n* = 5). Finally, adaptations targeting the socio‐political level (17%, *n* = 11) were predominantly motivated by societal/cultural norms (82%, *n* = 9).

### 3.6 Relationship to fidelity

None of the proposed adaptations impacted the core elements of the intervention (i.e. step assignment and step re‐assessment), therefore, all adaptations made by providers to optimize DiSC implementation at their facility were considered fidelity consistent.

### 3.7 Decision on adaptation implementation

Eighty‐three percent of adaptations were carried out as planned, with providers making the final decision to adopt (63%), adapt (35%) and abandon (2%) proposed adaptations. Only one adaptation, related to improving workflow by applying stickers to client files to make it easier to identify AYLHIV enrolled in the study, was abandoned. This adaptation was abandoned due to lack of supplies.

### 3.8 Implementation outcomes: acceptability, appropriateness and feasibility

**Figure 2:**
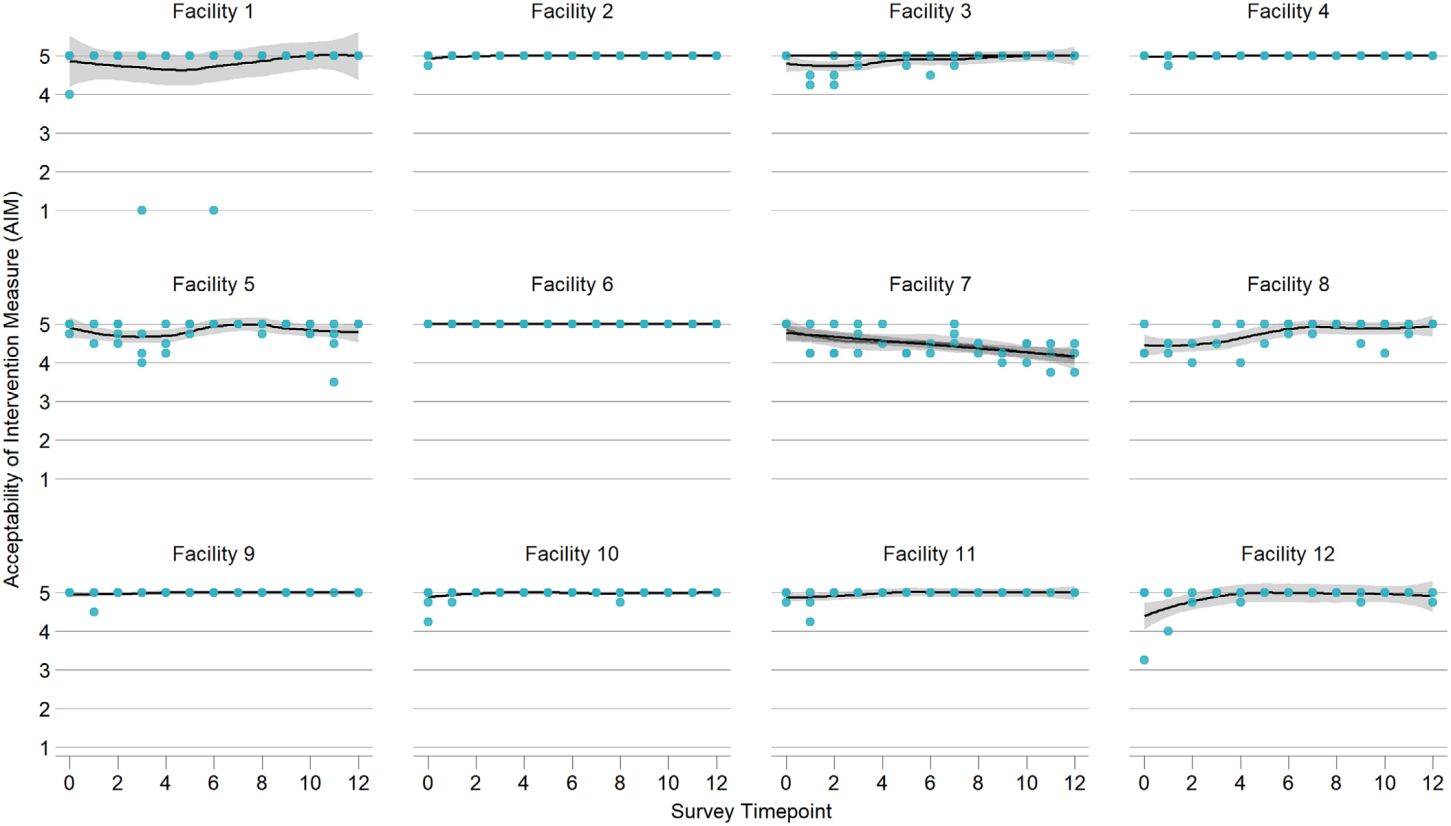
**Acceptability of Intervention Measure (AIM). Provider perceptions of acceptability during Stepped Care early implementation phase, including pre‐trial survey timepoint (April–December 2022)**.

**Figure 3:**
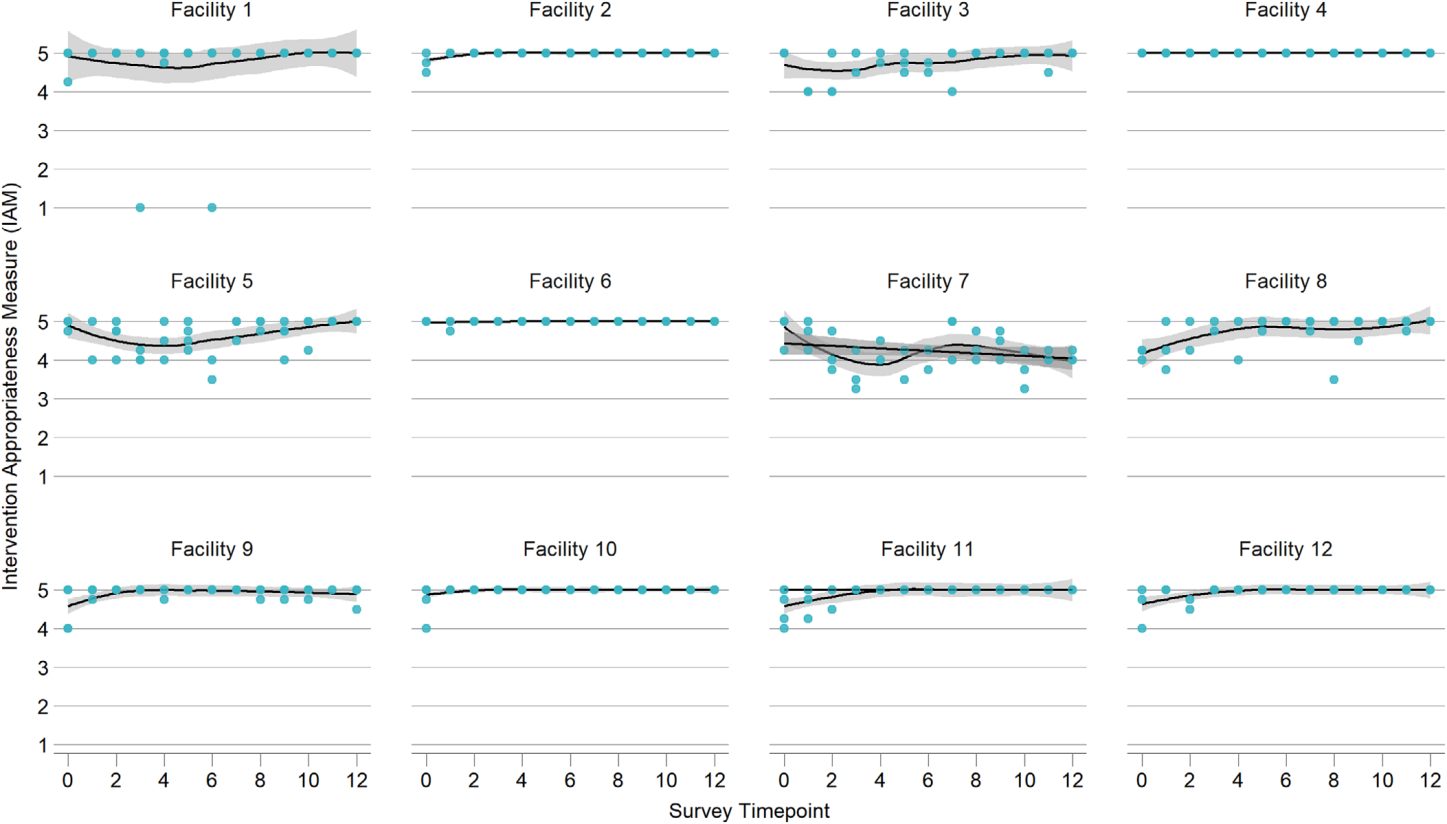
**Intervention Appropriateness Measure (IAM). Provider perceptions of appropriateness during Stepped Care early implementation phase, including pre‐trial survey timepoint (April–December 2022)**.

**Figure 4:**
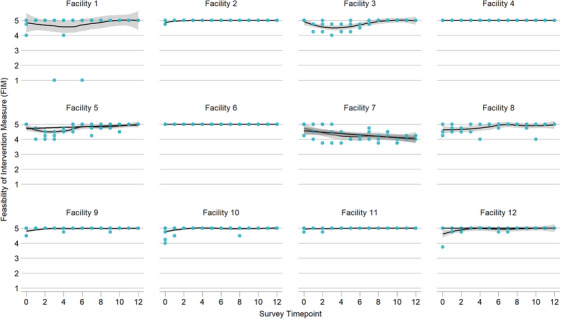
**Feasibility of Intervention Measure (FIM). Provider perceptions of feasibility during Stepped Care early implementation phase, including pre‐trial survey timepoint (April–December 2022)**.

Overall provider perceptions of acceptability, appropriateness and feasibility were high; trends were consistent across facilities and across early stage implementation outcome measures (Figures 2, 3, 4). Excluding pre‐trial scores, intervention acceptability, appropriateness and feasibility measures were consistently high throughout the implementation process (acceptability mean [SD]: 4.89 [0.35], median [IQR]: 5 [5, 5]; appropriateness mean [SD]: 4.85 [0.41], median [IQR]: 5 [5, 5]; feasibility mean [SD]: 4.87 [0.38], median [IQR]: 5 [5, 5]). However, provider perception did slightly improve over time. Average monthly change over time was statistically significantly higher across all implementation outcome measures (acceptability β = 0.011, 95% CI: 0.002, 0.020, *p* = 0.016; appropriateness β = 0.012, 95% CI: 0.007, 0.027, *p* < 0.001; feasibility β = 0.013, 95% CI: 0.004, 0.022, *p* = 0.005) (Table 3).

**Table 3:** Mixed effects linear regression model of health provider perception of acceptability, appropriateness and feasibility measures over time

	Acceptability			Appropriateness			Feasibility		
	β	95% CI	*p*‐value	β	95% CI	*p*‐value	β	95% CI	*p*‐value
Intercept	4.82	4.71, 4.93	–	4.75	4.60, 4.90	–	4.79	4.66, 4.93	–
Time (months)	0.011	0.002, 0.020	0.016	0.012	0.007, 0.027	<0.001	0.013	0.004, 0.022	0.005

Abbreviation: CI, confidence interval.

## 4 DISCUSSION

Intervention adaptability is a potential facilitator of implementation success and determinant‐informing strategies for scale‐up and scale‐out to new settings and populations [37]. Our analysis characterized provider‐identified adaptations across 12 HIV care clinics in western Kenya using FRAME, an adaptation framework previously applied across multiple disciplines, populations and settings [38, 39, 40, 41, 42, 43, 44, 45, 46, 47, 48, 49, 50, 51]. FRAME uses a systematic approach to understand how adaptations improve contextual fit, and improve relevance of intervention content. In our study, systematic tracking of adaptations enabled a better understanding of adaptation rationale and how to optimize intervention fit. Providers identified and made adaptations that addressed the challenges they experienced during implementation, such as incorporating the stepping tool into existing workflows. A key finding was that despite differences between clinic settings and organizational structures, providers made similar types of adaptations to improve intervention fit. With time, providers identified fewer challenges to address, and adaptations made had less impact on implementation outcomes since previous adaptations had already partially optimized the fit of DiSC to their setting. Understanding context during implementation informs applicability across settings by improving our knowledge of what is generalizable and what is not. We found that the majority of adaptations providers made were context‐specific, addressing clinic workflow and documentation challenges. A few adaptations focused on content, specifically adjusting for the school calendar.

The majority of provider adaptations were intended to align DiSC delivery to better meet AYLHIV needs and preferences, with minimal disruption to routine service provision. During implementation, context adaptations were made to ensure that administration of the PHQ‐2 depression screening tool, and adherence or CBT counselling sessions, were done in settings that provided privacy and confidentiality. For younger AYLHIV, providers adapted delivery to accommodate caregiver involvement as needed. Some AYLHIV preferred having caregivers present during clinic visits. Especially for younger AYLHIV (ages 10–14), providers found that caregiver involvement made it easier to explain the importance of keeping scheduled clinic appointments and deliver some components of DiSC, including CBT counselling sessions. Many adaptations were related to aligning clinic visit schedules to accommodate AYLHIV who were in boarding schools, common for adolescents and young adults in Kenya. We found that Step 1 of the DiSC intervention, differentiated care, was best aligned with the school calendar, which may be a motivating factor for AYLHIV to adhere to medications and routinely attend scheduled clinic visits in order to qualify for differentiated care visit schedules.

Adaptations that optimize intervention fit in real‐world settings can impact fidelity [26]. All adaptations made by providers during implementation were fidelity consistent and did not impact “core” components of the DiSC intervention, which included initial stepping with the clinical assessment tool, re‐stepping with the tool at each subsequent visit or receiving services aligned with the four care “steps” (differentiated care, standard of care, individual counselling or intensive support). The main content change made to the DiSC intervention was related to visit frequencies for different steps.

FRAME was originally designed and applied within the US‐based healthcare system with psychosocial interventions focused on mental health, substance abuse, sexual risk prevention and HIV prevention [24, 52]. As FRAME is used for different types of evidence‐based interventions and in different health systems contexts, it may need minor modifications. A potential future refinement of FRAME could be to connect the goal of an adaptation to service, client or implementation science outcomes [23]. Additionally, although FRAME categorizes the goal of an adaptation, it does not identify the potential relationship between an adaptation and the potential mechanism of action (and mediating or moderating effects) [53, 54] that brings about the desired outcome. Understanding potential mechanisms of action may inform the identification of implementation strategies to overcome challenges experienced during early phase implementation as well as future scale‐up.

Our study had several strengths. We collected quantitative measures of provider perceptions of acceptability, appropriateness and feasibility of the DiSC intervention over time [25]. These implementation outcome measures are important for monitoring successful implementation efforts. Giving providers support to identify challenges of importance to them and develop their own strategies to address them, may have contributed to improved perceptions of these measures over time. To our knowledge, simultaneously quantifying and tracking implementation outcomes alongside the characterization of adaptations is an innovation unique to our study.

Our analysis has limitations. Given our focus on adaptations to address intervention delivery, our analysis did not include evaluation of client‐reported outcomes. Although including quantitative measures of acceptability, appropriateness and feasibility was a strength of our study, it was also a limitation because these scores were consistently high throughout implementation. However, we demonstrated in the mixed effects linear model that provider perceptions slightly increased over time. We are not aware of alternative measures for these constructs that have evidence of reliability and validity. We note that facility 7, in comparison to other sites, showed a decrease in these measures over time. We did not identify any differences in participation in the CQI meetings related to cadres, numbers of meetings and types of adaptations made at this site. Overall, because providers made single or multiple adaptations at a time, it was challenging to link short‐term impact on improvement indicators of reach, adoption and fidelity with the adaptation being tested. It was also unclear whether observed impacts on outcomes are due to a single adaptation or the cumulative effect of several adaptations implemented sequentially. Additionally, reach and adoption may be a result of AYLHIV return to clinic schedule, and providers on leave or transferred out, respectively, rather than a result of the adaptation being tested. However, using a framework to unify adaptation descriptions is a first step towards understanding how adaptations impact implementation outcomes and success.

## 5 CONCLUSIONS

We used FRAME as a systematic way to track and categorize adaptations made to a multi‐component intervention supporting AYLHIV with retention in care. Adaptations identified by providers targeted improved integration into routine clinic practices and better meeting the needs of AYLHIV. Our study successfully adapted the elements of FRAME to capture adaptations to intervention delivery in HIV clinics in Kenya. Understanding adaptations within this framework was critical in identifying implementation challenges that were unique for AYLHIV services, such as the school calendar. The lessons learned during early phase implementation may inform Kenya Ministry of Health best practices for engaging youth and supporting them along the treatment and care continuum, ultimately informing future scalability of youth‐centred interventions [20] in HIV care clinics across Kenya.

## COMPETING INTERESTS

The authors have no competing interests to disclose.

## AUTHORS’ CONTRIBUTIONS

PKK, GJ‐S and KA serve as principal investigators of the study. JB, JK, WJ and KA contributed to data collection and management. DO provided support supervision for the quality improvement meetings. NC and DO categorized the adaptations data. NC analysed data with support from DO, KB‐S, WJ and BAR. NC drafted the initial manuscript. KB‐S, DIM, PKK, KA, BAR and GJ‐S critically reviewed and revised the manuscript. All authors have reviewed and approved the final manuscript.

## FUNDING

The research described in this publication was supported by the Eunice Kennedy Shriver National Institute of Child Health and Human Development through the PATC3H consortium (UG3/UH3 HD096906 [PKK, GJ‐S]) and F31HD105513 [NC]) of the National Institutes of Health, and the University of Washington CFAR (P30 AI027757).

## DISCLAIMER

The content is solely the responsibility of the authors and does not necessarily represent the official views of the National Institutes of Health.

6

## CME STATEMENT

This article is published as part of a supplement supported by unrestricted educational grant by ViiV Healthcare.

Credits Available for this Activity: American Medical Association (AMA Credit).

Washington University School of Medicine in St. Louis designates this enduring material for a maximum of 1 AMA PRA Category 1 Credit™. Physicians should claim only the credit commensurate with the extent of their participation in the activity.

## Data Availability

Data are available upon reasonable request.
